# Transcultural nursing care, religion, and compassion: A holistic path toward reduced inequalities

**DOI:** 10.1590/1980-220X-REEUSP-2025-0413en

**Published:** 2026-02-16

**Authors:** Ebru Dığrak, Ayfer Tezel

**Affiliations:** 1 Izmir University of Economics, Faculty of Health Sciences, Department of Nursing, Izmir, Türkiye.; 2 Ankara University, Faculty of Nursing, Ankara, Türkiye.

**Keywords:** Cultural Competency, Self-Compassion, Religion, Health Equity, Socioeconomic Factors, Education, Pharmacy, Competência Cultural, Autocompaixão, Religião, Equidade em Saúde, Fatores Socioeconômicos, Educação em Farmácia

## Abstract

**Objective:**

To examine the relationships between cultural competence, compassion, and religion among nursing students.

**Method:**

A cross-sectional study was conducted among third and fourth-year nursing students enrolled in a state university in Türkiye during the 2022–2023 academic year. A total of 422 students who met the inclusion criteria and agreed to participate completed the Personal Information Form, Individual Religion Inventory, Compassion Scale, and Cultural Competence Assessment Tool (CCATool). Data were analyzed using descriptive statistics, correlation analyses, and group comparison tests.

**Results:**

Gender was found to significantly influence cultural competence, compassion, and religion scores. Additionally, cultural competence was negatively correlated with both compassion and religion among the participating students.

**Conclusions:**

The findings highlight a concerning area within nursing education, emphasizing the need to enhance cultural competence alongside fostering compassion and religion. These results indicate the importance of developing nursing curricula that support quality education and contribute to reduced inequalities in healthcare delivery.

## RESUMO

**Objetivo:**

Examinar as relações entre competência cultural, compaixão e religião entre estudantes de enfermagem.

**Métodos:**

Foi realizado um estudo transversal entre estudantes do terceiro e quarto ano de enfermagem matriculados em uma universidade estadual na Turquia durante o ano letivo de 2022-2023. Um total de 422 alunos que atendiam aos critérios de inclusão e concordaram em participar preencheram o Formulário de Informações Pessoais, o Inventário Religioso Individual, a Escala de Compaixão e a Ferramenta de Avaliação de Competência Cultural (CCATool). Os dados foram analisados utilizando estatísticas descritivas, análises de correlação e testes de comparação de grupos.

**Resultados:**

Verificou-se que o gênero influenciava significativamente as pontuações relativas à competência cultural, compaixão e religião. Além disso, a competência cultural apresentou correlação negativa com a compaixão e a religião entre os alunos participantes.

**Conclusão:**

Os resultados destacam uma área preocupante na formação em enfermagem, enfatizando a necessidade de aprimorar a competência cultural, além de promover a compaixão e a religião. Esses resultados indicam a importância de desenvolver currículos de enfermagem que apoiem uma educação de qualidade e contribuam para a redução das desigualdades na prestação de cuidados de saúde.

## INTRODUCTION

At the global level, migration is primary driven by conflicts, economic instability, and social inequities, while population displacement is further intensified by the accelerating effects of climate change such as droughts, floods, and extreme weather events. The World Health Organization (WHO) underscores the necessity of strengthening health systems to effectively address the health needs of migrants and displaced populations affected by climate-related crises^(1)^. As of 2025, Türkiye hosts a substantial foreign population, including 1,069,397 individuals with residence permits, 2,829,266 Syrians under temporary protection, and approximately 9,000 persons under international protection status^(2)^. The large influx of refugees has not only increased the risk of communicable diseases but has also placed significant pressure on healthcare resources and logistics. To mitigate this burden, refugee health centers have been established to provide free healthcare services. Alongside standard healthcare personnel, these centers employ additional support staff, including social workers and interpreters, to address the specific needs of refugees^(3)^. Despite these efforts, migrants in Türkiye continue to face various barriers to healthcare access. Reported obstacles include discrimination, their distrust of healthcare professionals, cultural differences, limited knowledge of the healthcare system, and language barriers due to a lack of interpreters^(4)^.

In this context, it is crucial to enhance nurses’ cultural competence to ensure the delivery of effective and equitable care to patients from diverse cultural backgrounds. Cultural competence is a dynamic process encompassing the knowledge, attitudes, values, beliefs, behaviors, and skills required for effective communication with individuals, families, groups, and communities from diverse cultural backgrounds. Its primary aim is to provide high-quality, safe, accessible, and evidence-based nursing care while integrating intercultural interactions^(5)^. This recognition is closely linked to the concept of compassion, which involves proactive actions aimed at understanding and alleviating the suffering of others^(6)^. By integrating cultural competence with compassionate practice, nurses can foster respectful, empathetic, and culturally sensitive interactions, ensuring that care is not only clinically effective but also ethically and emotionally attuned to patients’ needs^(7)^.

Among the factors that can deepen the relationship between cultural competence and compassion are religion and religious values. In particular, Islam, the dominant religion in the country where the study was conducted, regards compassion and helping others as significant virtues, thereby fostering a strong sense of solidarity among individuals^(8)^. Dinham^(9)^ emphasizes that, in health and social environments, the diversity of religion and belief is interwoven with issues of globalization and migration. By promoting respect and understanding for cultural diversity, religion facilitates bridge-building between different cultures^(10)^. In this context, religion provides a significant framework that shapes interpersonal relationships, ultimately reinforcing the concept of cultural competence through its teachings. Thus, religion, compassion, and cultural competence play vital roles in fostering peace, empathy, and understanding, not only in interpersonal relations, but also at the societal level.

Moreover, from a cultural standpoint, religion has the potential to create a contradictory structure. While religiosity can sometimes pave the way for ethnocentric approaches disconnected from culture, it can also foster inclusive and community-oriented positive relationships^(11)^. These inconsistencies can affect compassion, a central element of religion, leading to a complex dynamic. This study aims to contribute to the understanding of these inconsistencies and to examine the relationship between three factors: cultural competence, religion, and compassion. Additionally, it seeks to provide important insights into the evaluation of religion and compassion, two key factors which influence cultural competence. From this perspective, there is currently no research that investigates the combined effect of three variables: cultural competence, compassion, and religion. Thus, the study aims to highlight the importance of discussing strategies aimed at developing these three conceptsin promoting societal understanding.

### Research Questions

1. Is there a relationship between cultural competence and compassion in nursing students?
2. Is there a relationship between cultural competence and religion nursing students?
3. What are the factors affecting levels of cultural competence, compassion, and religion in nursing students?

## METHOD

### Design of Study

This study employed a cross-sectional design to examine the relationships between cultural competence, compassion, and religion among nursing students.

### Study Population and Sample

The population of the study consisted of third-and fourth-year nursing students (N = 458) enrolled in the Nursing Department of a state university in Türkiye during the spring semester of the 2022–2023 academic year. Rather than using a sampling method, all students who met the inclusion criteria were invited to participate. The study was completed with 422 students who met the inclusion criteria (participation rate: 92%).

### Selection Critea

Students included in the study were 18 years of age or older, enrolled in the third or fourth year of the nursing program, and voluntarily agreed to participate in the research. Students were excluded if they did not speak Turkish, were on leave for any reason, or did not agree to participate. During the data collection process, 36 students either declined participation, could not be reached, or submitted incomplete forms.

### Data Collection Tools

The research data were collected using the Personal Information Form, Individual Religion Inventory, Compassion Scale, and Cultural Competence Assessment Tool (CCATool) – Student Version.

The Personal Information Form prepared by the researcher consisted of five questions regarding sociodemographic characteristics, grade, age, gender, family structure, and geographical region where he/she spent most of his/her life.

The Cultural Competence Assessment Tool (CCATool) – Student Version was developed based on the Papadopoulos Model to assess cultural competence. It comprises four sub-dimensions: cultural awareness, cultural knowledge, cultural sensitivity, and cultural practice. Each of these dimensions consists of ten items and is evaluated using a four-point Likert scale, with responses ranging from 1 (completely disagree) to 4 (completely agree). Following the administration of the scale, the calculated score distribution is used to determine the scores. Each correctly answered item (3 = Agree, 4 = Completely agree) scores one point, while incorrectly answered items (1 = Completely disagree, 2 = Disagree) score zero. The maximum total score for each dimension is 10; therefore, the maximum possible is 40. Additionally, the scoring system delineates four levels of cultural competence development: Incompetence, Awareness, Safety, and Competence. In the original study of the scale, the Cronbach’s alpha coefficient was determined to be greater than 0.80 for both the scale and the subscales^(12)^. In the Turkish validity and reliability study, a Cronbach alpha value of 0.87 was obtained^(13)^. In this study, the Cronbach alpha reliability value was determined to be 0.91.

The Compassion Scale (CS) developed by Pommier^(14)^ to assess individuals’ levels of compassion encompasses six sub-dimensions: Kindness, Indifference, Comman humanity, Separation, Mindfulness, and Disengagemnet. It consists of a total of 24 items evaluated on a five-point Likert scale (5 = Always, 4 = Often, 3 = Sometimes, 2 = Rarely, 1 = Never). Turkish validity and reliability study of this scale was conducted by Akdeniz and Deniz^(15)^ with university students. In that study, the Cronbach’s alpha value for the scale was found to be 0.85 and sub-dimensions ranged from 0.64 to 0.73. Similarly, in the present research, the Cronbach’s alpha value for the scale was determined 0.89 and sub-dimensions ranged from 0.70 to 0.78.

The Individual Religion Inventory (IRI) developed by Zagumny et al.^(16)^ aims to assess individuals’ levels of religiosity. It evaluates the influence of religion in individuals’ personal lives, their level of religion knowledge, the importance placed on developing their religion understanding, and the value assigned to religion in their lives. The scale consists of a single dimension and includes six items, rated using a five-point Likert scale, with the following options: (1 = Very untrue for me, 2 = Untrue for me, 3 = Somewhat true of me, 4 = True of me, 5 = Very true of me). Possible scores range from 6 to 30, higher scores are associated with greater levels of religious belief. Turkish reliability and validity study conducted by Ayten in 2013 resulted in a Cronbach’s alpha value of 0.851^(17)^. In the current study, the Cronbach’s alpha was 0.92.

### Data Collection

Data collection was conducted after obtaining permission from the school administration and faculty members, after the course schedules had been published. Data collection was conducted face-to-face in classroom settings during designated course hours. The research team explained the purpose and procedures of the study, obtained written informed consent, and assisted participants as needed. Completing the instruments required approximately 15–20 minutes. Potential sources of bias include self-selection bias due to voluntary participation and social desirability bias in self-reported measures. To mitigate these, anonymity was assured, and participants were informed that responses would be confidential and used solely for research purposes.

### Data Analysis and Treatment

Data analysis was performed using IBM SPSS Statistics version 26 (IBM Corp., Armonk, NY, USA). Descriptive statistics, including frequency, percentage, mean, and standard deviation, were used to summarize the sociodemographic characteristics. The normality of continuous variables was assessed using the Shapiro–Wilk and Kolmogorov–Smirnov tests, as well as Skewness and Kurtosis values. Pearson correlation analysis enabled examination of the relationships between continuous variables. The Student’s t-test was used to compare mean differences between two independent groups, while one-way analysis of variance (ANOVA) was applied for comparisons involving more than two groups. When statistically significant differences were detected in ANOVA, post-hoc pairwise comparisons were performed using the Bonferroni correction. A significance level of p < 0.05 was considered for all statistical tests. Only complete cases with fully answered data collection forms were included in the final analysis.

### Ethical Aspect

Prior to the commencement of the study, ethical approval was obtained from the Ethics Committee of Ankara University (Approval Date: 24/05/2021; Approval No: 08/96), as well as permission from the Faculty of Nursing at Ankara University. After providing the participants with detailed information about the study, written informed consent was obtained in accordance with the principles of the Declaration of Helsinki.

## RESULTS

Table 1 shows nursing the students’ sociodemographic characteristics and scale scores. Among the students, 82.5% are female, 57.6% are in their third year, and 80.5% come from nuclear family backgrounds. Additionally, 45.5% live in the central part of the country, 15.4% reside in the northern region, and 15.0% in the western region. The average age is 21.39 ± 0.88 years, with a range from 20 to 24 years (Table 1).

**Table 1:** Comparison of nursing students’ socio-demographic characteristics and scale scores – Ankara, Türkiye, 2023

	n (%)	Cultural competence assessment tool M ± SD	Cultural awareness M ± SD	Cultural knowledge M ± SD	Cultural sensitivity M ± SD	Cultural practice M ± SD	Compassion scale M ± SD	Individual religion inventory M ± SD
Grade	
3rd year	243 (57.6)	2.05 ± 0.25	1.76 ± 0.38	2.15 ± 0.22	2.52 ± 0.24	1.80 ± 0.35	4.09 ± 0.46	3.48 ± 0.52
4th year	179 (42.6)	1.96 ± 0.31	1.71 ± 0.37	1.96 ± 0.31	2.53 ± 0.25	1.80 ± 0.39	4.01 ± 0.49	3.43 ± 0.56
Statistics		.052	.181	.051	.650	.982	.337	.097
Gender	
Female	348 (82.5)	1.98 ± 0.27	1.71 ± 0.37	2.11 ± 0.22	2.49 ± 0.24	1.76 ± 0.33	4.11 ± 0.45	3.51 ± 0.53
Male	74 (17.5)	2.16 ± 0.27	1.87 ± 0.40	2.24 ± 0.23	2.70 ± 0.16	1.99 ± 0.46	3.80 ± 0.54	3.19 ± 0.51
Statistics		.000^**^	.000^**^	.000^**^	.000^**^	.000^**^	.000^**^	.000^**^
Family structure	
Nuclear	321 (76.1)	2.00 ± 0.28	1.72 ± 0.38	2.13 ± 0.23	2.51 ± 0.49	1.78 ± 0.35	4.10 ± 0.46	3.48 ± 0.54
Extended	102 (23.9)	2.06 ± 0.27	1.77 ± 0.38	2.15 ± 0.22	2.58 ± 0.24	1.85 ± 0.41	3.92 ± 0.50	3.37 ± 0.53
Statistics		.049^*^	.333	.490	.009^**^	.117	.087	.002^**^
Geographical region of longest residence	
Middle	192 (45.5)	1.91 ± 0.28	1.65 ± 0.33	2.09 ± 0.21	2.50 ± 0.25	1.72 ± 0.33	4.11 ± 0.44	3.55 ± 0.57
East	65 (15.4)	2.08 ± 0.21	1.90 ± 0.40	2.10 ± 0.23	2.50 ± 0.22	1.88 ± 0.29	4.11 ± 0.42	3.40 ± 0.40
North	63 (15.0)	2.09 ± 0.24	1.71 ± 0.38	2.23 ± 0.21	2.46 ± 0.27	1.74 ± 0.35	4.09 ± 0.48	3.45 ± 0.50
South	52 (12.3)	2.07 ± 0.27	1.78 ± 0.38	2.21 ± 0.22	2.65 ± 0.20	1.87 ± 0.44	3.83 ± 0.55	3.22 ± 0.59
West	50 (11.8)	2.13 ± 0.30	1.86 ± 0.39	2.17 ± 0.22	2.64 ± 0.21	1.97 ± 0.45	3.93 ± 0.52	3.41 ± 0.47
Statistics		.000^**^	.000^**^	.000^**^	.000^**^	.000^**^	.000^**^	.000^**^

Notes: M = Mean; SD = Standard deviation; Minimum = Min; Maximum; n = Number; % = Percent.

In the study, no statistically significant differences were found between students’ class levels and their scores on the cultural competence, religion, and compassion scales. However, a significant statistical difference was noted between scores and gender. Male students showed significantly higher scores in cultural competence and its subdimensions, while female students scored significantly higher on the religion and compassion scales (p < 0.01). Furthermore, there was a statistically significant difference between family type and the scores for cultural competence, the cultural sensitivity subdimension, and the religion scales. Students from extended families had significantly higher scores in both cultural competence (p < 0.05) and the cultural sensitivity subdimension (p < 0.001). In contrast, students from nuclear families scored significantly higher on the religion scale (p < 0.01). Additionally, the region in which students had spent the greater part of their lives exhibited statistically significant differences in their scores for cultural competence, religion, and compassion scales (p < 0.01) (Table 1).

Table 2 presents the average scores of nursing students on the cultural competence, religion, and compassion scales. The overall average score for cultural competence among the nursing students is 78.01 ± 13.04. The average score for religion is 22.17 ± 5.03, while the average score for compassion is 97.07 ± 12.18.

**Table 2:** Mean of the cultural competence assessment tool, compassion scale and individual religion inventory scores – Ankara, Türkiye, 2023

	M	SD	Min	Max	CA
*Cultural Competence Assessment Tool*	78.01	13.04	40	160	0.91
Cultural Awareness	16.65	4.69	10	40	0.88
Cultural Knowledge	19.28	4.12	10	40	0.77
Cultural Sensitivity	25.23	3.11	10	40	0.70
Cultural Practice	16.84	4.66	10	40	0.89
*Compassion Scale*	97.07	12.18	24	120	0.89
Kindness	16.24	2.65	4	20	0.76
Indifference	16.37	2.81	4	20	0.78
Common humanity	16.17	2.35	4	20	0.70
Separation	8.05	3.01	4	20	0.71
Mindfulness	7.95	2.87	4	20	0.71
Disengagement	7.69	2.80	4	20	0.72
*Individual Religion Inventory*	22.17	5.03	6	30	0.92

Notes: M = Mean; SD = Standard deviation; Minimum = Min; Maximum = Max; CA = Cronbach's alpha.

The correlations among cultural competence, religion, and compassion are presented in Table 3. The analysis indicates that nursing students’ scores in cultural competence and its subdimensions show a significant negative correlation with both religion and compassion (p < 0.05).

**Table 3:** Correlations between cultural competence assessment tool, compassion scale and individual religion inventory scores – Ankara, Türkiye, 2023

Variables	Compassion scale	Individual religion inventory
*Cultural Competence Assessment Tool*	-.198^**^	-.102^*^
Cultural Awareness	-.236^**^	-.150^**^
Cultural Knowledge	-.151^**^	-.174^**^
Cultural Sensitivity	-.315^**^	-.132^**^
Cultural Practice	-.263^**^	-.162^**^

Notes: * p < 0.05;

** p < 0.01 (two-tailed).

## DISCUSSION

Due to its geographical location, the region where the study was conducted hosts a significant number of refugees, while also being a popular tourist destination. Thus, nurses frequently encounter patients from diverse cultural backgrounds, and it is essential for nurses to provide culturally competent care to meet these patients’ specific needs. Our study has revealed that the cultural competence level among nursing students is below average, incontrast to previous research on this professional group in Türkiye, which indicated moderate to high levels of cultural competence^(13,18)^. In addition, a study covering nine countries reported a moderate level of cultural competence^(19)^. These findings highlight significant disparities in cultural competence both between countries and across different regions of the same country. One of the most likely reasons for these variations is differences in nursing education. The impact of these curricular differences on cultural competence levels is an important topic that warrants further research.

This study found a high level of compassion among nursing students. Similar findings have been reported in previous research on nursing students in Türkiye^(20,21,22)^. Literature from various countries, ranging from Iran and the UK, indicates moderate levels of compassion^(23,24,25)^. Among reasons for these differences, the most important is likely to be variations in the educational systems and the cultural diversity of the sample groups. Additionally, a contributing factor to the high level of compassion may be that 82.5% of the students in the study are women, who typically possess more advanced social and emotional skills.

The results of the study indicate a relatively high the level of religion among nursing students, aligning with previous research conducted on this population^(21,26)^. Despite the limited educational focus on religion topics within nursing education, the high level of religion belief is noteworthy. While there is a common perception that the younger generation is becoming increasingly distanced from religion^(26)^, it can be stated that university students demonstrate an above average level of commitment in this regard. This finding suggests that religious beliefs still hold significant importance among the young.

When examining the results of the three scales by gender, male students exhibited higher levels of cultural competence and its subdimensions, whereas female students scored higher on compassion and religion scales. A review of the literature similarly indicates a tendency towards higher cultural competence in males, and higher compassion levels in females^(20,22)^. A meta-analysis of 177 studies focusing on the relationship between gender and religion found that being female moderately increases levels of religion^(27)^. While there is general gender-specific characteristics, social and cultural influences are additional fundamental factors that shape individuals’ attitudes, and modifythese gender specific characteristics. It is generally considered that women’s roles as mothers, along with their emotional dispositions, cultural education, and social responsibilities, contribute to higher levels of compassion. Consequently, women’s emotional and empathetic tendencies may exceed those of men, and their traits of sharing and helping in communication align closely with concepts of cooperation and sharing found in religions, making them more inclined to a positive attitude toward religious practices. Moreover, a study found that, compared to female nurses, males demonstrated higher confidence levels when interacting with patients from diverse cultural backgrounds^(28)^; consequently, it is suggested that females may lag behind in areas of cultural competence such as cultural curiosity and communication security. This disparity points to the need for targeted educational strategies to empower female nursing students in enhancing their cultural competence.

This study identified a significant negative correlation between cultural competence and compassion among nursing students, while in a study conducted by Nobahar et al.^(23)^ no significant relationship was found. Conversely, research conducted in the United States revealed a significant relationship between compassion and cultural competence, indicating that cultural competence serves as a predictor of compassion^(29)^. The current study contributes new insights to the relationship between cultural competence and compassion. The presence of a negative correlation suggests that lower levels of cultural competence may be associated with reduced sensitivity toward individuals from diverse backgrounds, or more generally, with taking a different approach to the social and emotional factors that shape compassion. This relationship between cultural competence and compassion emerges as a critical area to consider in the design of educational curricula for nursing students.

This study demonstrates a significant negative correlation between cultural competence and religion among nursing students. However, the results are mixed. In a similar investigation, a positive statistical correlation was found between the level of religion and intercultural sensitivity^(15)^, on the other hand, in another study, religion/spiritual beliefs were found not to significantly impact the cultural competence of healthcare providers^(30)^. Another research effort focusing on religion and global citizenship revealed a paradox in which religion simultaneously contributes to societal values and diminishes them^(10)^. In a study examining how students’ religious beliefs affect their attitudes to ethnicity, some reported that religious teachings negatively impacted their perspectives on various ethnic groups, leading toprejudice against different cultural groups, while others indicated that such teachings promote the idea of equality among all^(31)^. As seen, the results in the literature regarding the relationship between religion and attitudes toward different cultures remains mixed and inadequate. New insights from this study suggests that even with positive attitudes towards religion, a lack of cultural competence may hinder the development of a positive outlook towards diverse cultures. Therefore, it provides a significant contribution to the literature by indicating that even relatively strong feelings of religion and compassion may be undrermined by a lack of cultural competence, and consequently, have no meaningful positive impact.

These findings align with the United Nations Sustainable Development Goals, particularly Goal 3 (Good Health and Well-Being), Goal 4 (Quality Education), and Goal 10 (Reduced Inequalities)^(32)^. The low level of cultural competence among students indicates the need to strengthen cultural awareness and intercultural communication training within nursing education. The high levels of compassion and religion are positive characteristics; nevertheless, the integration of these values into inclusive care practices requires the systematic incorporation of cultural competence into educational processes. Accordingly, restructuring nursing curricula to include a culturally sensitive and equity-oriented approach may help reduce inequalities in healthcare services and contribute to improving overall public health.

### Limitations

This study is significant as the first to explore nursing students’ levels of cultural competence, compassion, and religiosity, as well as the relationships among these. The data presented has the potential to serve as a valuable resource for developing new strategies aimed at enhancing cultural competence within nursing education programs. By focusing on these critical dimensions, this study contributes to existing literature and emphasizes the importance of preparing nursing students to address the diverse needs of patients in a multicultural healthcare environment.

Despite its strengths, the study has several limitations. It was conducted within a single nursing department at one university in Türkiye, which may restrict the generalizability of the findings. Additionally, due to its cross-sectional design, the study cannot establish causal relationships among the variables, which limits the ability to infer the direction of these relationships. Furthermore, the reliance on self-reported structured surveys may introduce bias, and the responses may not fully capture the depth of nursing students’ understanding of cultural competence, compassion, and religion factors. These limitations warrant caution in interpreting the results and underscore the need for further research to validate the findings across diverse contexts and populations.

## CONCLUSION

This study revealed that nursing students demonstrated lower-than-expected cultural competence levels, but relatively higher compassion and religiosity levels. Additionally, male students were found to have higher cultural competence scores, and female students, higher levels of compassion and religiosity. A significant negative relationship was identified between cultural competence and both compassion and religiosity, indicating that greater emotional or religious sensitivity does not necessarily translate into culturally competent behavior.

Based on these findings, it is essential to strengthen cultural competence training within nursing education programs. Students can be encouraged to develop culturally responsive care skills through the incorporation of case-based discussions, simulations, and experiential learning activities related to cultural diversity into the curriculum. To ensure higher levels of compassion in clinical practice, reflective practice sessions and empathy-based learning experiences should be integrated into training. Additionally, addressing the influence of religiosity within an ethical and professional framework can support students in understanding how personal beliefs may shape care decisions. To reduce gender-based differences, for male students, educational strategies should focus on enhancing compassion-based communication skills among and, for female students, on strengthening cultural competence. Accordingly, integrating the development of cultural competence and compassion skills through educational policies and curriculum design will enhance the capacity of future nurses to provide fair, effective, and culturally sensitive care to diverse communities.

## Data Availability

The dataset supporting the findings of this study is not publicly available, however, the full dataset supporting the findings of this study is available upon request to the corresponding author.

## References

[1] World Health Organization. (2025). Health system strengthening interventions to improve the health of displaced and migrant populations in the context of climate change [Internet].. https://www.who.int/publications/i/item/9789240112452.

[2] Presidency of Migration Management. (2025). Presidency of migration management, up-to-date statistics [Internet].. https://en.goc.gov.tr/.

[3] Ekmekci PE (2017). Syrian Refugees, health and migration legislation in Turkey.. J Immigr Minor Health.

[4] Barış M, Sert G, Önder O (2025). Ethical challenges in accessing and providing healthcare for Syrian refugees in Türkiye.. Bioethics.

[5] Andrews M, Boyle J (2016). Transcultural concepts in nursing care..

[6] Papadopoulos I (2018). Culturally competent compassion: a guide for healthcare students and practitioners..

[7] Hernandez NC, Leal LMR, Brito MJM (2022). Building culturally competent compassion in nurses caring for vulnerable populations.. J Holist Nurs.

[8] Karakaya M (2019). Erdem ahlakindaki erdemler ile islam ahlakindaki güzel davranışlar arasindaki uyum.. Agri islâmi ilimler Dergisi.

[9] Dinham A (2018). Religion and belief in health and social care: the case for religious literacy.. Int J Hum Rights Healthc.

[10] Millar C, Chahda L, Carey LB, Ly A, McLaren PO, Drakopoulos E (2024). Global citizenship: Cultural, religious and spiritual-an exploratory scoping review.. J Relig Health.

[11] Katzarska-Miller I, Barnsley CA, Reysen S (2014). Global citizenship identification and religiosity.. Arch Psychol Relig.

[12] Papadopoulos I (2006). Transcultural health and social care: development of culturally competent practitioners..

[13] Dığrak E, Tezel A (2022). Validity and reliability of the Turkish Version of the Cultural Competence Assessment Tool in nursing students.. Clin Exp Health Sci.

[14] Pommier EA (2011). The compassion scale.. Diss Abstr Int A Humani Soc Sci.

[15] Akdeniz S, Deniz ME (2016). Merhamet Ölçeği’nin Türkçeye uyarlanmasi: geçerlik ve güvenirlik çaliımasi.. J Happiness Well-Being.

[16] Zagumny MJ, Pierce KE, Adams K, Fallos SL (2012). Presentation at the 24th Annual Convention.

[17] Ayten A (2013). Din ve sağlik: bireysel dindarlik, sağlik davranışlari ve hayat memnuniyeti ilişkisi üzerine bir araştirma.. Din Bilimleri Akademik Araıtirma Dergisi.

[18] Özdemir S, Sevinç S (2023). Correlation between cultural competence, xenophobia, and attitudes towards brain drain in nursing students.. Nurse Educ Today.

[19] Cruz JP, Aguinaldo AN, Estacio JC, Alotaibi A, Arguvanli S, Cayaban ARR (2018). A multicountry perspective on cultural competence among baccalaureate nursing students.. J Nurs Scholarsh.

[20] Bilgiç § (2022). Does the compassion level of nursing students affect their ethical sensitivity?. Nurse Educ Today.

[21] Birimoglu Okuyan C, Akgul E, Gurcay B (2024). The relationship between religious attitude, compassion and spiritual care in nursing students: the case of Türkiye.. J Relig Health.

[22] Kavradim ST, Akgün M, Ozer Z, Boz İ (2019). Perception of compassion and professional values in nursing students: a cross-sectional multivariate analysis from Turkey.. Nurse Educ Pract.

[23] Nobahar M, Yarahmadi S, Raiesdana N, Delshad ES, Hajizadegan F, Ebrahimzadeh F (2022). Predicting moral intelligence in nursing students and its relationships with self-compassion, and cultural competence: a cross-sectional study.. BMC Nurs.

[24] Kotera Y, Conway E, Van Gordon W (2019). Mental health of UK university business students: relationship with shame, motivation and self-compassion.. J Educ Bus.

[25] Edoho Samson-Akpan P, Lee Y, Baqer Al-Jubouri M, Rose Cayaban A, John ME (2022). Compassion competence among nursing students from different cultures: a multinational study.. J Nurs Educ.

[26] Düzgüner S, Sevinc K (2020). The relationship between altruism and religious attitude among university students from different departments.. Theosophia.

[27] Doğan M, Karaca F (2021). Cinsiyetin dindarlik üzerine etkisi: bir meta analiz çaliçmasi.. Bilimname.

[28] Kargin M, Çiftçi MÇ (2020). Bir üniversite hastanesinde klinikte çalıçan hemçirelerin kültürlerarasi duyarliliginin belirlenmesi.. Turkiye Klinikleri J Nurs Sci.

[29] Gottlieb M, Shibusawa T (2020). The impact of self-compassion on cultural competence: results from a quantitative study of MSW students.. J Soc Work Educ.

[30] Saadi A, Taleghani S, Dillard A, Ryan G, Heilemann M, Eisenman D (2023). Original research: nurses’ experiences with racial, ethnic, cultural, and religious discrimination in the workplace: a qualitative study.. Am J Nurs.

[31] Sağir Z, Paloutzian RF (2020). Religious attitudes, demographic variables, and prejudice toward syrian refugees in Turkey: local data and international differences.. J Middle East Migr Stud.

[32] United Nations. (2015). The Sustainable Development Goals [Internet].. https://www.un.org/sustainabledevelopment/development-goals/.

