# Supplementary material for: Transcultural nursing care, religion, and compassion: A holistic path toward reduced inequalities
Source: Rev Esc Enferm USP. 2026 Feb 16;60:e20250413. doi: 10.1590/1980-220X-REEUSP-2025-0413en (PMC12930534; doi:10.1590/1980-220X-REEUSP-2025-0413en)
Supplement: Translation

# Translation: Cuidados de enfermería transculturales, religión y compasión: Un camino holístico hacia la reducción de las desigualdades

**DOI:** 10.1590/1980-220X-REEUSP-2025-0413es

**Authors:** Ebru Dığrak, Ayfer Tezel

**Affiliations:** 1 Izmir University of Economics, Faculty of Health Sciences, Department of Nursing, Izmir, Türkiye.; 2 Ankara University, Faculty of Nursing, Ankara, Türkiye.

**Keywords:** Competencia Cultural, Autocompasión, Religión, Equidad en Salud, Factores Socioeconómicos, Educación en Farmacia

## Abstract

**Objetivo:**

Examinar las relaciones entre la competencia cultural, la compasión y la religión entre los estudiantes de enfermería.

**Método:**

Se realizó un estudio transversal entre estudiantes de tercer y cuarto año de enfermería matriculados en una universidad estatal de Turquía durante el año académico 2022-2023. Un total de 422 estudiantes que cumplían los criterios de inclusión y aceptaron participar completaron el formulario de información personal, el inventario religioso individual, la escala de compasión y la herramienta de evaluación de la competencia cultural (CCATool). Los datos se analizaron mediante estadísticas descriptivas, análisis de correlación y pruebas de comparación de grupos.

**Resultados:**

Se descubrió que el género influía significativamente en las puntuaciones de competencia cultural, compasión y religión. Además, la competencia cultural se correlacionó negativamente con la compasión y la religión entre los estudiantes participantes.

**Conclusión:**

Los resultados ponen de relieve un aspecto preocupante dentro de la formación en enfermería, haciendo hincapié en la necesidad de mejorar la competencia cultural, además de fomentar la compasión y la religión. Estos resultados indican la importancia de desarrollar planes de estudios de enfermería que respalden una educación de calidad y contribuyan a reducir las desigualdades en la prestación de asistencia sanitaria.

## INTRODUCCIÓN

A nivel mundial, la migración está impulsada principalmente por los conflictos, la inestabilidad económica y las desigualdades sociales, mientras que el desplazamiento de la población se ve intensificado por los efectos acelerados del cambio climático, como las sequías, las inundaciones y los fenómenos meteorológicos extremos. La Organización Mundial de la Salud (OMS) subraya la necesidad de fortalecer los sistemas de salud para atender eficazmente las necesidades sanitarias de los migrantes y las poblaciones desplazadas afectadas por crisis relacionadas con el clima^(1)^. En 2025, Turquía acoge a una importante población extranjera, que incluye 1 069 397 personas con permiso de residencia, 2 829 266 sirios bajo protección temporal y aproximadamente 9000 personas bajo protección internacional^(2)^. La gran afluencia de refugiados no solo ha aumentado el riesgo de enfermedades transmisibles, sino que también ha ejercido una presión significativa sobre los recursos y la logística sanitarios. Para mitigar esta carga, se han creado centros de salud para refugiados que prestan servicios sanitarios gratuitos. Además del personal sanitario habitual, estos centros cuentan con personal de apoyo adicional, como trabajadores sociales e intérpretes, para atender las necesidades específicas de los refugiados^(3)^. A pesar de estos esfuerzos, los migrantes en Turquía siguen enfrentándose a diversas barreras para acceder a la atención sanitaria. Entre los obstáculos señalados figuran la discriminación, la desconfianza hacia los profesionales sanitarios, las diferencias culturales, el conocimiento limitado del sistema sanitario y las barreras lingüísticas debido a la falta de intérpretes^(4)^.

En este contexto, es fundamental mejorar la competencia cultural de las enfermeras para garantizar la prestación de una atención eficaz y equitativa a pacientes de diversos orígenes culturales. La competencia cultural es un proceso dinámico que abarca los conocimientos, actitudes, valores, creencias, comportamientos y habilidades necesarios para comunicarse de manera eficaz con personas, familias, grupos y comunidades de diversos orígenes culturales. Su objetivo principal es proporcionar una atención de enfermería de alta calidad, segura, accesible y basada en la evidencia, al tiempo que se integran las interacciones interculturales^(5)^. Este reconocimiento está estrechamente relacionado con el concepto de compasión, que implica acciones proactivas destinadas a comprender y aliviar el sufrimiento de los demás^(6)^. Al integrar la competencia cultural con la práctica compasiva, las enfermeras pueden fomentar interacciones respetuosas, empáticas y culturalmente sensibles, garantizando que la atención no solo sea clínicamente eficaz, sino también ética y emocionalmente acorde con las necesidades de los pacientes^(7)^.

Entre los factores que pueden profundizar la relación entre la competencia cultural y la compasión se encuentran la religión y los valores religiosos. En particular, el islam, la religión dominante en el país donde se realizó el estudio, considera la compasión y la ayuda a los demás como virtudes importantes, lo que fomenta un fuerte sentido de solidaridad entre las personas^(8)^. Dinham^(9)^ destaca que, en los entornos sanitarios y sociales, la diversidad de religiones y creencias está entrelazada con cuestiones de globalización y migración.

Al promover el respeto y la comprensión de la diversidad cultural, la religión facilita la construcción de puentes entre diferentes culturas^(10)^. En este contexto, la religión proporciona un marco significativo que da forma a las relaciones interpersonales, reforzando en última instancia el concepto de competencia cultural a través de sus enseñanzas. Así, la religión, la compasión y la competencia cultural desempeñan un papel fundamental en el fomento de la paz, la empatía y la comprensión, no solo en las relaciones interpersonales, sino también a nivel social.

Además, desde un punto de vista cultural, la religión tiene el potencial de crear una estructura contradictoria. Si bien la religiosidad puede a veces allanar el camino para enfoques etno-céntricos desconectados de la cultura, también puede fomentar relaciones positivas inclusivas y orientadas a la comunidad^(11)^. Estas inconsistencias pueden afectar a la compasión, un elemento central de la religión, lo que da lugar a una dinámica compleja. Este estudio tiene como objetivo contribuir a la comprensión de estas inconsistencias y examinar la relación entre tres factores: la competencia cultural, la religión y la compasión. Además, pretende aportar información importante para la evaluación de la religión y la compasión, dos factores clave que influyen en la competencia cultural. Desde esta perspectiva, actualmente no existe ninguna investigación que estudie el efecto combinado de tres variables: la competencia cultural, la compasión y la religión. Por lo tanto, el estudio pretende destacar la importancia de debatir estrategias destinadas a desarrollar estos tres conceptos para promover la comprensión social.

### Preguntas de Investigación

1. Existe una relación entre la competencia cultural y la compasión en los estudiantes de enfermería?
2. Existe una relación entre la competencia cultural y la religión en los estudiantes de enfermería?
3. Cuáles son los factores que afectan a los niveles de competencia cultural, compasión y religión en los estudiantes de enfermería?

## MÉTODO

### Diseño del Estudio

Este estudio empleó un diseño transversal para examinar las relaciones entre la competencia cultural, la compasión y la religión entre los estudiantes de enfermería.

### Población y Muestra del Estudio

La población del estudio estuvo compuesta por estudiantes de tercer y cuarto año de enfermería (N = 458) matriculados en el Departamento de Enfermería de una universidad estatal de Turquía durante el semestre de primavera del año académico 2022-2023. En lugar de utilizar un método de muestreo, se invitó a participar a todos los estudiantes que cumplían los criterios de inclusión. El estudio se completó con 422 estudiantes que cumplían los criterios de inclusión (tasa de participación: 92%).

### Criterios de Selección

Los estudiantes incluidos en el estudio tenían 18 años o más, estaban matriculados en el tercer o cuarto año del programa de enfermería y aceptaron voluntariamente participar en la investigación. Se excluyó a los estudiantes que no hablaban turco, estaban de baja por cualquier motivo o no aceptaron participar. Durante el proceso de recopilación de datos, 36 estudiantes rechazaron participar, no pudieron ser localizados o enviaron formularios incompletos.

### Herramientas de Recopilación de Datos

Los datos de la investigación se recopilaron utilizando el Formulario de Información Personal, el Inventario de Religión Individual, la Escala de Compasión y la Herramienta de Evaluación de la Competencia Cultural (Compassion Scale, and Cultural Competence Assessment Tool-CCATool), versión para estudiantes.

El Formulario de Información Personal elaborado por el investigador constaba de cinco preguntas relativas a las características sociodemográficas, el grado, la edad, el género, la estructura familiar y la región geográfica en la que había pasado la mayor parte de su vida.

La Herramienta de Evaluación de la Competencia Cultural (CCATool) – Versión para estudiantes se desarrolló basándose en el Modelo Papadopoulos para evaluar la competencia cultural. Comprende cuatro subdimensiones: conciencia cultural, conocimiento cultural, sensibilidad cultural y práctica cultural. Cada una de estas dimensiones consta de diez ítems y se evalúa utilizando una escala Likert de cuatro puntos, con respuestas que van desde 1 (totalmente en desacuerdo) hasta 4 (totalmente de acuerdo).

Tras la administración de la escala, se utiliza la distribución de puntuaciones calculada para determinar las puntuaciones. Cada ítem respondido correctamente (3 = de acuerdo, 4 = totalmente de acuerdo) obtiene un punto, mientras que los ítems respondidos incorrectamente (1 = totalmente en desacuerdo, 2 = en desacuerdo) obtienen cero puntos. La puntuación máxima total para cada dimensión es 10; por lo tanto, la máxima posible es 40. Además, el sistema de puntuación delimita cuatro niveles de desarrollo de la competencia cultural: incompetencia, conciencia, seguridad y competencia. En el estudio original de la escala, se determinó que el coeficiente alfa de Cronbach era superior a 0,80 tanto para la escala como para las subescalas^(12)^. En el estudio de validez y fiabilidad turco, se obtuvo un valor alfa de Cronbach de 0,87^(13)^. En este estudio, se determinó que el valor de fiabilidad alfa de Cronbach era de 0,91.

La Escala de Compasión (Compassion Scale – CS) desarrollada por Pommier^(14)^ para evaluar los niveles de compasión de las personas abarca seis subdimensiones: amabilidad, indiferencia, humanidad común, separación, atención plena y desvinculación. Consta de un total de 24 ítems evaluados en una escala Likert de cinco puntos (5 = Siempre, 4 = A menudo, 3 = A veces, 2 = Rara vez, 1 = Nunca). El estudio de validez y fiabilidad turco de esta escala fue realizado por Akdeniz y Deniz^(15)^ con estudiantes universitarios. En ese estudio, se determinó que el valor alfa de Cronbach para la escala era de 0,85 y que las subdimensiones oscilaban entre 0,64 y 0,73. De manera similar, en la presente investigación, se determinó que el valor alfa de Cronbach para la escala era de 0,89 y que las subdimensiones oscilaban entre 0,70 y 0,78.

El Inventario de Religión Individual (IRI) desarrollado por Zagumny et al.^(16)^ tiene como objetivo evaluar los niveles de religiosidad de las personas. Evalúa la influencia de la religión en la vida personal de las personas, su nivel de conocimiento religioso, la importancia que conceden al desarrollo de su comprensión de la religión y el valor que asignan a la religión en sus vidas. La escala consta de una sola dimensión e incluye seis ítems, calificados mediante una escala Likert de cinco puntos, con las siguientes opciones: (1 = Muy falso para mí, 2 = Falso para mí, 3 = Algo cierto para mí, 4 = Cierto para mí, 5 = Muy cierto para mí). Las puntuaciones posibles oscilan entre 6 y 30, y las puntuaciones más altas se asocian con mayores niveles de creencia religiosa. El estudio de fiabilidad y validez realizado en Turquía por Ayten en 2013 dio como resultado un valor alfa de Cronbach de 0,851^(17)^. En el presente estudio, el alfa de Cronbach fue de 0,92.

### Recopilación de Datos

La recopilación de datos se llevó a cabo tras obtener el permiso de la administración de la escuela y los miembros del cuerpo docente, una vez publicados los horarios de las clases. La recopilación de datos se realizó de forma presencial en las aulas durante las horas lectivas designadas. El equipo de investigación explicó el propósito y los procedimientos del estudio, obtuvo el consentimiento informado por escrito y ayudó a los participantes según fue necesario. La cumplimentación de los instrumentos requirió aproximadamente entre 15 y 20 minutos. Las posibles fuentes de sesgo incluyen el sesgo de autoselección debido a la participación voluntaria y el sesgo de deseabilidad social en las medidas autoinformadas. Para mitigarlos, se garantizó el anonimato y se informó a los participantes de que las respuestas serían confidenciales y se utilizarían únicamente con fines de investigación.

### Análisis y Tratamiento de Datos

El análisis de datos se realizó utilizando IBM SPSS Statistics versión 26 (IBM Corp., Armonk, NY, EE. UU.). Se utilizaron estadísticas descriptivas, incluyendo frecuencia, porcentaje, media y desviación estándar, para resumir las características sociodemográficas. La normalidad de las variables continuas se evaluó utilizando las pruebas de Shapiro-Wilk y Kolmogorov-Smirnov, así como los valores de asimetría y curtosis. El análisis de correlación de Pearson permitió examinar las relaciones entre las variables continuas.

Se utilizó la prueba t de Student para comparar las diferencias medias entre dos grupos independientes, mientras que se aplicó un análisis de varianza unidireccional (ANOVA) para las comparaciones que involucraban a más de dos grupos. Cuando se detectaron diferencias estadísticamente significativas en el ANOVA, se realizaron comparaciones por pares post hoc utilizando la corrección de Bonferroni. Se consideró un nivel de significación de p < 0,05 para todas las pruebas estadísticas. Solo se incluyeron en el análisis final los casos completos con los formularios de recopilación de datos totalmente respondidos.

### Aspectos Éticos

Antes del inicio del estudio, se obtuvo la aprobación ética del Comité de Ética de la Universidad de Ankara (fecha de aprobación: 24/05/2021; n.º de aprobación: 08/96), así como el permiso de la Facultad de Enfermería de la Universidad de Ankara. Tras proporcionar a los participantes información detallada sobre el estudio, se obtuvo el consentimiento informado por escrito de acuerdo con los principios de la Declaración de Helsinki.

## RESULTADOS

La Tabla 1 muestra las características sociodemográficas de los estudiantes de enfermería y las puntuaciones de la escala. Entre los estudiantes, el 82.5% son mujeres, el 57.6% están en su tercer año y el 80.5% provienen de familias nucleares. Además, el 45.5% vive en la parte central del país, el 15.4% reside en la región norte y el 15.0% en la región oeste. La edad media es de 21,39 ± 0,88 años, con un rango de 20 a 24 años (Tabla 1).

**Tabla 1:** Comparación de las características sociodemográficas y las puntuaciones de la escala de los estudiantes de enfermería – Ankara, Turquía, 2023

	n (%)	Herramienta de evaluación de la competencia cultural M ± SD	Conciencia cultural M ± SD	Conocimiento cultural M ± SD	Sensibilidad cultural M ± SD	Práctica cultural M ± SD	Escala de compasión M ± SD	Inventario de religión individual M ± SD
Curso	
3.o año	243 (57,6)	2,05 ± 0,25	1,76 ± 0,38	2,15 ± 0,22	2,52 ± 0,24	1,80 ± 0,35	4,09 ± 0,46	3,48 ± 0,52
4.o año	179 (42,6)	1,96 ± 0,31	1,71 ± 0,37	1,96 ± 0,31	2,53 ± 0,25	1,80 ± 0,39	4,01 ± 0,49	3,43 ± 0,56
Estadísticas		0,052	0,181	0,051	0,650	0,982	0,337	0,097
Género	
Mujer	348 (82,5)	1,98 ± 0,27	1,71 ± 0,37	2,11 ± 0,22	2,49 ± 0,24	1,76 ± 0,33	4,11 ± 0,45	3,51 ± 0,53
Hombre	74 (17,5)	2,16 ± 0,27	1,87 ± 0,40	2,24 ± 0,23	2,70 ± 0,16	1,99 ± 0,46	3,80 ± 0,54	3,19 ± 0,51
Estadísticas		0,000^**^	.000^**^	0,000^**^	.000^**^	.000^**^	0,000^**^	.000^**^
Estructura familiar	
Nuclear	321 (76,1)	2,00 ± 0,28	1,72 ± 0,38	2,13 ± 0,23	2,51 ± 0,49	1,78 ± 0,35	4,10 ± 0,46	3,48 ± 0,54
Extendida	102 (23,9)	2,06 ± 0,27	1,77 ± 0,38	2,15 ± 0,22	2,58 ± 0,24	1,85 ± 0,41	3,92 ± 0,50	3,37 ± 0,53
Estadísticas		0,049^*^	0,333	0,490	0,009^**^	0,117	0,087	0,002^**^
Región geográfica de residencia más prolongada	
Media	192 (45,5)	1,91 ± 0,28	1,65 ± 0,33	2,09 ± 0,21	2,50 ± 0,25	1,72 ± 0,33	4,11 ± 0,44	3,55 ± 0,57
Este	65 (15,4)	2,08 ± 0,21	1,90 ± 0,40	2,10 ± 0,23	2,50 ± 0,22	1,88 ± 0,29	4,11 ± 0,42	3,40 ± 0,40
Norte	63 (15,0)	2,09 ± 0,24	1,71 ± 0,38	2,23 ± 0,21	2,46 ± 0,27	1,74 ± 0,35	4,09 ± 0,48	3,45 ± 0,50
Sur	52 (12,3)	2,07 ± 0,27	1,78 ± 0,38	2,21 ± 0,22	2,65 ± 0,20	1,87 ± 0,44	3,83 ± 0,55	3,22 ± 0,59
Oeste	50 (11,8)	2,13 ± 0,30	1,86 ± 0,39	2,17 ± 0,22	2,64 ± 0,21	1,97 ± 0,45	3,93 ± 0,52	3,41 ± 0,47
Estadísticas		0,000^**^	0,000^**^	0,000^**^	0,000^**^	0,000^**^	0,000^**^	0,000^**^

Notas: M = media; SD = desviación estándar; mínimo = min; máximo; n = número; % = porcentaje.

En el estudio no se encontraron diferencias estadísticamente significativas entre los niveles de clase de los estudiantes y sus puntuaciones en las escalas de competencia cultural, religión y compasión. Sin embargo, se observó una diferencia estadística significativa entre las puntuaciones y el género. Los estudiantes varones obtuvieron puntuaciones significativamente más altas en competencia cultural y sus subdimensiones, mientras que las estudiantes mujeres obtuvieron puntuaciones significativamente más altas en las escalas de religión y compasión (p < 0,01). Además, hubo una diferencia estadísticamente significativa entre el tipo de familia y las puntuaciones en las escalas de competencia cultural, la subdimensión de sensibilidad cultural y la religión. Los estudiantes de familias extensas obtuvieron puntuaciones significativamente más altas tanto en competencia cultural (p < 0.05) como en la subdimensión de sensibilidad cultural (p < 0.001). Por el contrario, los estudiantes de familias nucleares obtuvieron puntuaciones significativamente más altas en la escala de religión (p < 0,01). Además, la región en la que los estudiantes habían pasado la mayor parte de sus vidas mostró diferencias estadísticamente significativas en sus puntuaciones en las escalas de competencia cultural, religión y compasión (p < 0,01) (Tabla 1).

La tabla 2 presenta las puntuaciones medias de los estudiantes de enfermería en las escalas de competencia cultural, religión y compasión. La puntuación media global de competencia cultural entre los estudiantes de enfermería es de 78,01 ± 13,04. La puntuación media de religión es de 22,17 ± 5,03, mientras que la puntuación media de compasión es de 97,07 ± 12,18.

**Tabla 2:** Media de las puntuaciones de la herramienta de evaluación de la competencia cultural, la escala de compasión y el inventario de religión individual – Ankara, Turquía, 2023

	M	SD	Mín	Máx	CA
*Herramienta de evaluación de la competencia cultural*	78,01	13,04	40	160	0,91
Conciencia cultural	16,65	4,69	10	40	0,88
Conocimientos culturales	19,28	4,12	10	40	0,77
Sensibilidad cultural	25,23	3,11	10	40	0,70
Práctica cultural	16,84	4,66	10	40	0,89
*Escala de compasión*	97,07	12,18	24	120	0,89
Amabilidad	16,24	2,65	4	20	0,76
Indiferencia	16,37	2,81	4	20	0,78
Humanidad común	16,17	2,35	4	20	0,70
Separación	8,05	3,01	4	20	0,71
Mindfulness	7,95	2,87	4	20	0,71
Desvinculación	7,69	2,80	4	20	0,72
*Inventario de religión individual*	22,17	5,03	6	30	0,92

Notas: M = media; SD = desviación estándar; mínimo = min; máximo = máx.; CA = alfa de Cronbach.

Las correlaciones entre la competencia cultural, la religión y la compasión se presentan en la tabla 3. El análisis indica que las puntuaciones de los estudiantes de enfermería en competencia cultural y sus subdimensiones muestran una correlación negativa significativa tanto con la religión como con la compasión (p < 0,05).

**Tabla 3:** Correlaciones entre la herramienta de evaluación de la competencia cultural, la escala de compasión y las puntuaciones del inventario de religión individual – Ankara, Turquía, 2023

Variables	Escala de compasión	Inventario de religión individual
*Herramienta de evaluación de la competencia cultural*	-0,198^**^	-0,102^*^
Conciencia cultural	-0,236^**^	-0,150^**^
Conocimiento cultural	-0,151^**^	-0,174^**^
Sensibilidad cultural	-0,315^**^	-0,132^**^
Práctica cultural	-0,263^**^	-0,162^**^

Notas: * p < 0,05;

** p < 0,01 (bilateral).

## DISCUSIÓN

Debido a su ubicación geográfica, la región donde se llevó a cabo el estudio acoge a un número significativo de refugiados, al tiempo que es un destino turístico muy popular. Por lo tanto, las enfermeras se encuentran con frecuencia con pacientes de diversos orígenes culturales, y es esencial que proporcionen una atención culturalmente competente para satisfacer las necesidades específicas de estos pacientes. Nuestro estudio ha revelado que el nivel de competencia cultural entre los estudiantes de enfermería está por debajo de la media, en contraste con investigaciones anteriores sobre este grupo profesional en Turquía, que indicaban niveles moderados a altos de competencia cultural^(13,18)^. Además, un estudio que abarcaba nueve países informó de un nivel moderado de competencia cultural^(19)^. Estos hallazgos ponen de relieve las importantes disparidades en materia de competencia cultural tanto entre países como entre diferentes regiones del mismo país.

Una de las razones más probables de estas variaciones son las diferencias en la formación de enfermería. El impacto de estas diferencias curriculares en los niveles de competencia cultural es un tema importante que merece una investigación más profunda. Este estudio encontró un alto nivel de compasión entre los estudiantes de enfermería. Se han reportado hallazgos similares en investigaciones previas sobre estudiantes de enfermería en Turquía^(20,21,22)^.

La bibliografía de varios países, desde Irán hasta el Reino Unido, indica niveles moderados de compasión^(23,24,25)^. Entre las razones de estas diferencias, la más importante es probablemente las variaciones en los sistemas educativos y la diversidad cultural de los grupos de muestra. Además, un factor que contribuye al alto nivel de compasión puede ser que el 82,5% de los estudiantes del estudio son mujeres, que suelen poseer habilidades sociales y emocionales más avanzadas.

Los resultados del estudio indican un nivel relativamente alto de religiosidad entre los estudiantes de enfermería, en consonancia con investigaciones anteriores realizadas sobre esta población^(21,26)^. A pesar del limitado enfoque educativo en temas religiosos dentro de la formación en enfermería, es notable el alto nivel de creencia religiosa. Si bien existe la percepción común de que la generación más joven se está alejando cada vez más de la religión^(26)^, se puede afirmar que los estudiantes universitarios muestran un nivel de compromiso superior a la media en este sentido.

Este hallazgo sugiere que las creencias religiosas siguen teniendo una importancia significativa entre los jóvenes. Al examinar los resultados de las tres escalas por género, los estudiantes varones mostraron niveles más altos de competencia cultural y sus subdimensiones, mientras que las estudiantes mujeres obtuvieron puntuaciones más altas en las escalas de compasión y religión. Una revisión de la literatura indica de manera similar una tendencia hacia una mayor competencia cultural en los varones y mayores niveles de compasión en las mujeres^(20,22)^.

Un metaanálisis de 177 estudios centrados en la relación entre género y religión reveló que ser mujer aumenta moderadamente los niveles de religión^(27)^. Si bien existen características generales específicas de cada género, las influencias sociales y culturales son factores fundamentales adicionales que configuran las actitudes de las personas y modifican estas características específicas de cada género. En general, se considera que el papel de las mujeres como madres, junto con su disposición emocional, su educación cultural y sus responsabilidades sociales, contribuyen a unos niveles más altos de compasión. En consecuencia, las tendencias emocionales y empáticas de las mujeres pueden superar a las de los hombres, y sus rasgos de compartir y ayudar en la comunicación se alinean estrechamente con los conceptos de cooperación y compartir que se encuentran en las religiones, lo que las hace más propensas a tener una actitud positiva hacia las prácticas religiosas. Además, un estudio reveló que, en comparación con las enfermeras, los enfermeros mostraban mayores niveles de confianza al interactuar con pacientes de diversos orígenes culturales^(28)^; en consecuencia, se sugiere que las mujeres pueden estar rezagadas en áreas de competencia cultural como la curiosidad cultural y la seguridad en la comunicación. Esta disparidad apunta a la necesidad de estrategias educativas específicas para empoderar a las estudiantes de enfermería en la mejora de su competencia cultural.

Este estudio identificó una correlación negativa significativa entre la competencia cultural y la compasión entre los estudiantes de enfermería, mientras que en un estudio realizado por Nobahar et al.^(23)^ no se encontró una relación significativa. Por el contrario, una investigación realizada en Estados Unidos reveló una relación significativa entre la compasión y la competencia cultural, lo que indica que la competencia cultural sirve como predictor de la compasión^(29)^. El presente estudio aporta nuevos conocimientos sobre la relación entre la competencia cultural y la compasión. La presencia de una correlación negativa sugiere que los niveles más bajos de competencia cultural pueden estar asociados con una menor sensibilidad hacia las personas de diversos orígenes o, de manera más general, con un enfoque diferente de los factores sociales y emocionales que conforman la compasión. Esta relación entre la competencia cultural y la compasión se perfila como un área crítica a tener en cuenta en el diseño de los planes de estudio para los estudiantes de enfermería.

Este estudio demuestra una correlación negativa significativa entre la competencia cultural y la religión entre los estudiantes de enfermería. Sin embargo, los resultados son contradictorios. En una investigación similar, se encontró una correlación estadística positiva entre el nivel de religión y la sensibilidad intercultural^(15)^, mientras que, en otro estudio, se observó que la religión y las creencias espirituales no tenían un impacto significativo en la competencia cultural de los profesionales de la salud^(30)^. Otra investigación centrada en la religión y la ciudadanía global reveló una paradoja en la que la religión contribuye simultáneamente a los valores sociales y los disminuye^(10)^. En un estudio que examinaba cómo las creencias religiosas de los estudiantes afectan a sus actitudes hacia la etnicidad, algunos informaron de que las enseñanzas religiosas influían negativamente en sus perspectivas sobre diversos grupos étnicos, lo que daba lugar a prejuicios contra diferentes grupos culturales, mientras que otros indicaron que dichas enseñanzas promueven la idea de igualdad entre todos^(31)^. Como se ha visto, los resultados de la literatura sobre la relación entre la religión y las actitudes hacia las diferentes culturas siguen siendo contradictorios e insuficientes. Las nuevas conclusiones de este estudio sugieren que, incluso con actitudes positivas hacia la religión, la falta de competencia cultural puede obstaculizar el desarrollo de una visión positiva hacia las diversas culturas. Por lo tanto, supone una contribución significativa a la literatura al indicar que incluso los sentimientos relativamente fuertes de religión y compasión pueden verse socavados por la falta de competencia cultural y, en consecuencia, no tener un impacto positivo significativo.

Estos hallazgos se alinean con los Objetivos de Desarrollo Sostenible de las Naciones Unidas, en particular el Objetivo 3 (Buena salud y bienestar), el Objetivo 4 (Educación de calidad) y el Objetivo 10 (Reducción de las desigualdades)^(32)^. El bajo nivel de competencia cultural entre los estudiantes indica la necesidad de reforzar la formación en materia de conciencia cultural y comunicación intercultural dentro de la educación en enfermería. Los altos niveles de compasión y religión son características positivas; sin embargo, la integración de estos valores en las prácticas de atención inclusiva requiere la incorporación sistemática de la competencia cultural en los procesos educativos. En consecuencia, la reestructuración de los planes de estudio de enfermería para incluir un enfoque culturalmente sensible y orientado a la equidad puede ayudar a reducir las desigualdades en los servicios de salud y contribuir a mejorar la salud pública en general.

### Limitaciones

Este estudio es significativo por ser el primero en explorar los niveles de competencia cultural, compasión y religiosidad de los estudiantes de enfermería, así como las relaciones entre ellos. Los datos presentados pueden servir como un valioso recurso para desarrollar nuevas estrategias destinadas a mejorar la competencia cultural en los programas de formación en enfermería. Al centrarse en estas dimensiones críticas, este estudio contribuye a la bibliografía existente y destaca la importancia de preparar a los estudiantes de enfermería para atender las diversas necesidades de los pacientes en un entorno sanitario multicultural.

A pesar de sus puntos fuertes, el estudio tiene varias limitaciones. Se llevó a cabo en un solo departamento de enfermería de una universidad de Turquía, lo que puede limitar la generalización de los resultados. Además, debido a su diseño transversal, el estudio no puede establecer relaciones causales entre las variables, lo que limita la capacidad de inferir la dirección de estas relaciones. Por otra parte, el hecho de basarse en encuestas estructuradas autoinformadas puede introducir sesgos, y es posible que las respuestas no reflejen plenamente la profundidad de la comprensión de los estudiantes de enfermería sobre los factores de competencia cultural, compasión y religión. Estas limitaciones justifican la cautela a la hora de interpretar los resultados y subrayan la necesidad de seguir investigando para validar los hallazgos en diversos contextos y poblaciones.

## CONCLUSIÓN

Este estudio reveló que los estudiantes de enfermería mostraron niveles de competencia cultural inferiores a los esperados, pero niveles relativamente más altos de compasión y religiosidad. Además, se observó que los estudiantes varones tenían puntuaciones más altas en competencia cultural y las estudiantes mujeres, niveles más altos de compasión y religiosidad. Se identificó una relación negativa significativa entre la competencia cultural y tanto la compasión como la religiosidad, lo que indica que una mayor sensibilidad emocional o religiosa no se traduce necesariamente en un comportamiento culturalmente competente.

A partir de estos resultados, es esencial reforzar la formación en competencia cultural dentro de los programas de educación en enfermería. Se puede animar a los estudiantes a desarrollar habilidades de atención culturalmente sensibles mediante la incorporación al plan de estudios de debates basados en casos, simulaciones y actividades de aprendizaje experiencial relacionadas con la diversidad cultural. Para garantizar niveles más altos de compasión en la práctica clínica, se deben integrar en la formación sesiones de práctica reflexiva y experiencias de aprendizaje basadas en la empatía. Además, abordar la influencia de la religiosidad dentro de un marco ético y profesional puede ayudar a los estudiantes a comprender cómo las creencias personales pueden influir en las decisiones de atención. Para reducir las diferencias de género, en el caso de los estudiantes varones, las estrategias educativas deben centrarse en mejorar las habilidades de comunicación basadas en la compasión, y en el caso de las estudiantes mujeres, en fortalecer la competencia cultural. En consecuencia, la integración del desarrollo de la competencia cultural y las habilidades de compasión a través de políticas educativas y el diseño del plan de estudios mejorará la capacidad de los futuros enfermeros para proporcionar una atención justa, eficaz y culturalmente sensible a comunidades diversas.

## Data Availability

El conjunto de datos que respalda los resultados de este estudio no está disponible públicamente; sin embargo, el conjunto de datos completo que respalda los resultados de este estudio está disponible previa solicitud al autor correspondiente.
